# Anisotropic elasticity of silicon and its application to the modelling of X-ray optics

**DOI:** 10.1107/S1600577514004962

**Published:** 2014-04-04

**Authors:** Lin Zhang, Raymond Barrett, Peter Cloetens, Carsten Detlefs, Manuel Sanchez del Rio

**Affiliations:** a European Synchrotron Radiation Facility, 6 Rue Jules Horowitz, BP 220, 38043 Grenoble, France

**Keywords:** anisotropic elasticity of silicon, crystal orientation, thermal deformation, bent mirror, cryogenic cooled monochromator, anisotropic Poisson’s ratio

## Abstract

The crystal lattice of single-crystal silicon gives rise to anisotropic elasticity. The stiffness and compliance coefficient matrix depend on crystal orientation and, consequently, Young’s modulus, the shear modulus and Poisson’s ratio as well. Computer codes (in Matlab and Python) have been developed to calculate these anisotropic elasticity parameters for a silicon crystal in any orientation. These codes facilitate the evaluation of these anisotropy effects in silicon for applications such as microelectronics, microelectromechanical systems and X-ray optics. For mechanically bent X-ray optics, it is shown that the silicon crystal orientation is an important factor which may significantly influence the optics design and manufacturing phase. Choosing the appropriate crystal orientation can both lead to improved performance whilst lowering mechanical bending stresses. The thermal deformation of the crystal depends on Poisson’s ratio. For an isotropic constant Poisson’s ratio, ν, the thermal deformation (RMS slope) is proportional to (1 + ν). For a cubic anisotropic material, the thermal deformation of the X-ray optics can be approximately simulated by using the average of ν_12_ and ν_13_ as an effective isotropic Poisson’s ratio, where the direction 1 is normal to the optic surface, and the directions 2 and 3 are two normal orthogonal directions parallel to the optical surface. This average is independent of the direction in the optical surface (the crystal plane) for Si(100), Si(110) and Si(111). Using the effective isotropic Poisson’s ratio for these orientations leads to an error in thermal deformation smaller than 5.5%.

## Abstract 2

Anisotropic elasticity of single-crystal silicon, applications to modelling of a bent X-ray mirror, and thermal deformation of a liquid-nitrogen-cooled monochromator crystal are presented.

## 1. Introduction   

Single-crystal silicon is a perfect crystal which, owing to its interesting mechanical and physical properties, is widely used for X-ray optics at synchrotron light sources. Example applications include silicon crystal monochromators in both Bragg and Laue configurations, silicon substrates for high-heat-load white-beam mirrors, bent Kirkpatrick–Baez focusing mirrors and multilayer optics. It is well known that silicon is an anisotropic material whose mechanical properties, such as elastic modulus *E*, Poisson’s ratio ν and shear modulus *G*, depend on the orientation of the crystal lattice. The anisotropic stiffness coefficients for the (100) crystal plane of silicon have been initially determined by experiments (Mason, 1958 ▸; Wortman & Evans, 1965 ▸; Hall, 1967 ▸). Determination of the stiffness constants from these values for an arbitrary crystal orientation (*hkl*) therefore requires the use of the direction cosines referred to the crystal axis of the Si(100) orientation. Parameters such as Poisson’s ratio and shear modulus depend on two directions, and it is important to correctly take into account these crystallographic directions in the calculation of anisotropic elastic properties. Most previous modelling work on silicon-based X-ray optics has been performed using the simplifying assumption of isotropic material properties. A few studies have, however, taken into account the anisotropic material properties for bent diffracting crystals and reflecting mirrors. The anisotropic elasticity has been applied to study the X-ray reflectivity of doubly curved Bragg diffracting crystals (Chukhovskii *et al.*, 1994 ▸) and Laue crystals meridional (Schulze & Chapman, 1995 ▸) or sagittal (Zhong *et al.*, 2002 ▸) bending. Li & Khounsary (2004 ▸) considered the anisotropic Poisson’s ratio varying with direction in the silicon (100) crystal plane for the calculation of the anticlastic bending radius in bendable optics. Zhang (2010 ▸) presented matrix-based Matlab code for the calculation of the anisotropic elastic properties of silicon, and application to bendable mirror width profile optimization. This study showed the influence of the crystal orientation on the bending force and stress in the mirror. The anisotropic mechanical properties of silicon were also considered in thermal deformation analysis of liquid-nitrogen-cooled silicon crystals under high heat load (Zhang *et al.*, 2013 ▸).

Many existing synchrotron light sources (ESRF, APS, SPring-8,…) are planning and implementing significant facility upgrades, and some low-emittance synchrotron light sources (NSLS II, MAX IV,…) are under construction. The improved source characteristics of these light sources can only be fully utilized if the beamline performance and consequent specifications of optical components are pushed to higher levels than the current stage. Photon flux preservation, beam collimation, focusing and preservation of coherence are required for optical elements in the beamline. In the design and optimization of the beamline optics it is essential to have accurate and reliable predictions of the shape of the optical elements under high heat load or bending forces. For these purposes, the anisotropic elasticity should be considered in the modelling of the silicon-based optics.

For the high-heat-load X-ray optics, the anisotropic elasticity intervenes in the thermal stress through both Young’s modulus and Poisson’s ratio, but in the thermal deformation mainly through Poisson’s ratio.

In this paper we first report the anisotropic elasticity of single-crystal silicon and compare our results with some literature values. Then we apply these properties to a bent mirror substrate, and discuss the influence of crystal orientation on the bending forces, stress and on the mirror shape profile. Finally, we focus upon the thermal deformation modelling of silicon-based optics with anisotropic elasticity, and investigate the influence of Poisson’s ratio on the thermal deformation.

## 2. Anisotropic mechanical properties of the silicon   

**Figure 1:**
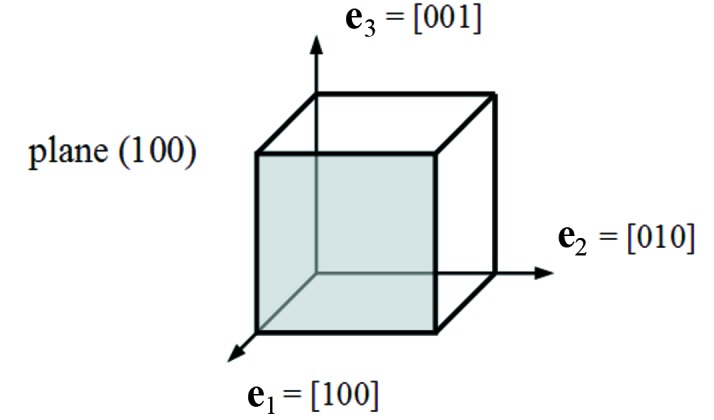
Conventional crystal-axis coordinate system for crystal plane (100).

The generalized Hooke’s law to express the relation between the stress and strain in a continuous elastic material can be written as (Nye, 1957 ▸; Hearmon, 1961 ▸)
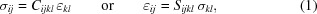
where *C*_*ijkl*_ and *S*_*ijkl*_ are, respectively, the stiffness and compliance fourth-rank tensors, and σ_*ij*_ and *∊*_*kl*_ are second-rank stress and strain tensors. Generally, the stiffness or compliance tensor has 81 elements. However, owing to the symmetry of the stress and strain tensors, and also the stiffness (or compliance) tensor, there are only 21 independent elastic coefficients in the stiffness (or compliance) tensor for a general anisotropic linear elastic solid. This reduction in number of independent coefficients makes it possible to simplify the notation and calculations by expressing the compliance and stiffness tensors in the form of 6 × 6 symmetric matrices, and the stress and strain tensors in the form of six-element vectors. Any pair of tensor indices *ij* (or equivalently *ji*) collapse into a single index. The most used notation in bibliography makes the assignment: 11 → 1, 22 → 2, 33 → 3, 23 → 4, 31 → 5 and 12 → 6. We can, for instance, contract terms *C*_1132_ to *C*_14_, ∊_31_ to ∊_5_, and σ_12_ to σ_6_. Silicon and germanium have the same cubic diamond crystal structure. The cubic lattice system consists of a set of three axes described by three lattice vectors orthogonal and of equal length. The conventional crystal-axis coordinate system for crystal plane (100) is defined by the normal vector **e**_1_ = [100] and two other orthogonal vectors in the crystal plane, **e**_2_ = [010] and **e**_3_ = [001], as shown in Fig. 1 ▸. In this conventional coordinate system, the stiffness coefficient matrix reduces to the following structure with only three independent elastic coefficients,
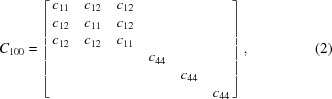
for Si(100), where *c*_11_ = 165.7, *c*_12_ = 63.9, *c*_44_ = 79.6 GPa (Mason, 1958 ▸). These coefficients are commonly used in the literature although Hall (1967 ▸) proposed data with slightly better accuracy (*c*_11_ = 165.6, *c*_12_ = 63.9, *c*_44_ = 79.5 GPa), but the difference between the two are not significant. In this paper, we use the data from Mason (1958 ▸) in order to make the comparison with some other studies.

The compliance matrix is the inverse of the stiffness matrix,
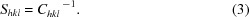
For Si(100), the compliance matrix *S*_100_ has the same structure as the stiffness matrix (2). The three independent coefficients are *s*_11_ = (*c*_11_ + *c*_12_)/[(*c*_11_ − *c*_12_)(*c*_11_ + 2*c*_12_)] = 7.68, *s*_12_ = −*c*_12_/[(*c*_11_ − *c*_12_)(*c*_11_ + 2*c*_12_)] = −2.14, *s*_44_ = 1/*c*_44_ = 12.56 × 10^−12^ Pa^−1^. For an arbitrary orientation of the cubic crystal with optical surface parallel to the (*h k l*) plane, a convenient coordinate system is to use the crystallographic orientations that are defined by how the crystal has been cut. This new coordinate system (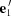, 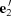, 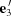) is defined by the surface normal [*h k l*] and two additional orthogonal vectors in the crystal surface. This choice is also valid for any asymmetrical crystal cutting which can be expressed *via* fractional *h k l* indices. Therefore, the vector 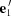 is along the surface normal direction [*h k l*], and vectors 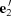 and 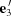 are parallel to the crystal surface (*h k l*). To determine the compliance matrix for this particular orientation, one can rotate the crystal-axis coordinate system (**e**_1_, **e**_2_, **e**_3_) to the new coordinate system (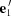, 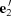, 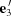). For instance, the normal vector is [*h k l*] and the two other orthogonal vectors in the plane could be [0 *l −k*], [(*k* ^2^ + *l* ^2^) − h*k − h*l] for (k*l ≠ 0). Therefore the normalized vectors are
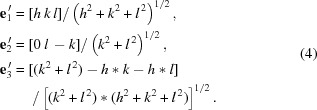
The formalism of Wortman & Evans (1965 ▸) can be used to calculate the stiffness coefficient matrix *C*_*hkl*_ and compliance matrix *S*_*hkl*_ for any silicon crystal orientation. For all classes of cubic crystals, Young’s modulus *E*_*hkl*_ in any crystallographic direction [*h k l*] can be calculated by the following equations (Nye, 1957 ▸; Wortman & Evans, 1965 ▸; Brantley, 1973 ▸),
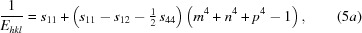
or, equivalently,
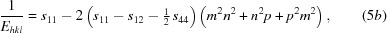
where *m*, *n*, *p* are the direction cosines for the direction along which *E* is calculated, and *s*_*ij*_ are the three independent elastic compliances referred to the crystal axes (Fig. 1 ▸), as defined by equation (3). Knowing the relation *m* ^2^ + *n* ^2^ + *p* ^2^ = 1, the transformation between (5*a*) and (5*b*) is straightforward. Here the direction cosines can be calculated by
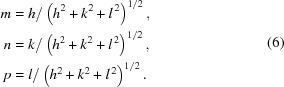
Young’s modulus *E*_*hkl*_ in the crystallographic direction [*h k l*] is independent of the choice of the coordinate system. For the particular directions [100], [110], [111] and [311], the above equations can be simplified as follows,
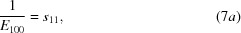


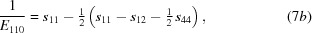


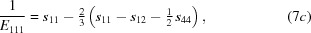


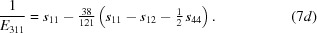
These equations give the results *E*_100_ = 130, *E*_110_ = 169, *E*_111_ = 188, *E*_311_ = 152 GPa. Note that the Young’s modulus along (111) is almost 45% larger than along (100). Poisson’s ratio and the shear modulus for an anisotropic crystal are given in general by (Nye, 1957 ▸, Wortman & Evans, 1965 ▸)
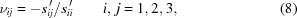


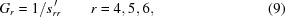
where 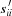 and 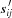 are the elastic compliance coefficients in the new coordinate system and vary with crystal orientation. The Poisson’s ratio ν_*ij*_ corresponds to the ratio of the strain variation (contraction) in the direction 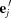 when a strain variation (extension) is applied in the direction 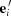. The shear modulus *G*_*r*=4_ = *G*_23_ (*G*_*r*=5_ = *G*_31_ and *G*_*r*=6_ = *G*_12_) represents the ratio of shear stress to the shear strain involving directions 23: 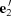 and 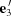 (31 and 12). For cubic crystals, equations (8) and (9) can be written as (Wortman & Evans, 1965 ▸; Brantley, 1973 ▸)
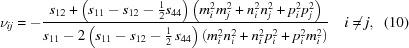


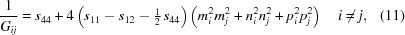
where (*m*_*i*_, *n*_*i*_, *p*_*i*_) and (*m*_*j*_, *n*_*j*_, *p*_*j*_) are the direction cosines for the 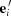 direction and 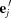 direction with respect to the crystal axes defined by Fig. 1 ▸.

**Figure 2:**
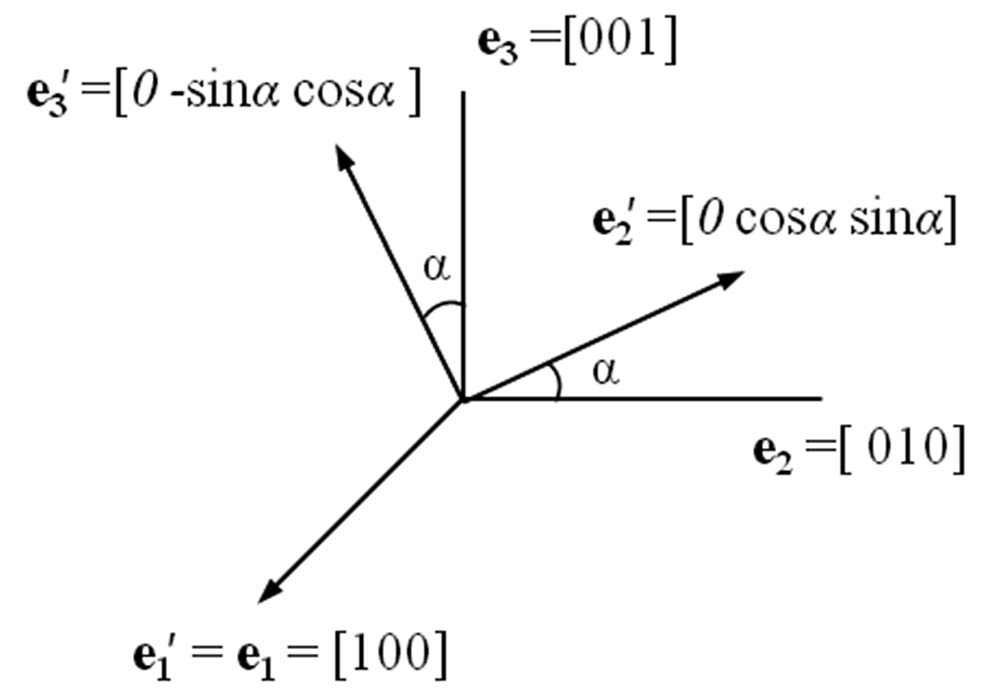
The crystal-axis coordinate system (**e**_1_, **e**_2_, **e**_3_) and the new coordinate system (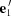, 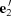, 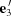) for crystal plane (100). The vector 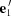 is fixed in the normal direction [100], and the vectors 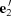 and 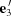 are in the crystal plane (100). The angle α is between the vectors 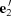 and **e**_2_.

It is very convenient to calculate the stiffness matrix *C*, compliance matrix *S*, Young’s modulus *E*, shear modulus *G* and Poisson’s ratio ν by using computer code. We wrote matrix-based Matlab code (Zhang, 2010 ▸) and also implemented in Python using NumPy; these codes are summarized in the supporting information.^1^ Using these codes we have performed calculations of Young’s modulus *E*, the shear modulus *G* and Poisson’s ratio ν for some commonly used silicon crystal orientations Si(100), (110), (111), (311). The new coordinate system (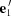, 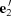, 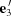) is defined as explained previously. As an example for Si(100): the vector 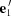 = **e**_1_ is in the surface normal direction [1 0 0], and the vectors 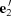 and 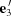 are in the crystal plane obtained by rotating an angle α of the initial crystal axes **e**_2_ and **e**_3_ as shown in Fig. 2 ▸.

**Figure 3:**
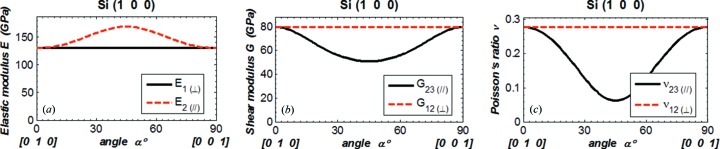
(*a*) Elastic modulus in the directions 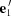 and 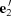. (*b*) Shear modulus and (*c*) Poisson’s ratio in the directions 12 and 23 for Si(100). The coordinate system is defined as shown in Fig. 2 ▸. The angle α is between the vectors 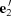 and [0 1 0] in the crystal plane: 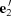
(α = 0°) = [0 1 0], 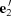
(α = 90°) = [0 0 1].

In order to compare our results with data in the literature (Wortman & Evans, 1965 ▸; Kim *et al.*, 2001 ▸; Hopcroft *et al.*, 2010 ▸), we plot Young’s modulus *E* in the direction 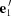 normal to the crystal plane (*E*_⊥_) and in the direction 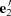 parallel to the crystal surface (*E*_∥_) *versus* angle α in Figs. 3(*a*) ▸ ▸ ▸–6(*a*) ▸, the shear modulus *G*_12(⊥)_ and *G*_23(∥)_ in Figs. 3(*b*) ▸–6(*b*), and Poisson’s ratio ν_12(⊥)_ and ν_23(∥)_ in Figs. 3(*c*) ▸–6(*c*) ▸. The values of *E*, *G* and ν are in the new coordinate system; the prime symbol (′) is omitted. The parallel symbol (∥) and perpendicular symbol (⊥) are used to indicate, respectively, the two orthogonal directions both parallel to the crystal plane, and the two orthogonal directions of which one is normal and the other is parallel to the crystal plane. When the angle α varies from 0° to 90°, the directions of the vector 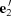 rotated 90° in the crystal plane are as shown in Figs. 3 ▸–6 ▸ by the vectors below the horizontal axis.

For Young’s modulus, results are given in the [*h k l*] direction 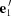 normal to the crystal (*h k l*) plane (⊥), and in directions 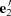 within the (*h k l*) crystal plane (∥). In the direction normal to the crystal plane, it is natural that Young’s modulus *E*_1(⊥)_ is independent of the direction in the plane (angle α) for all four cases as shown in Figs. 3 ▸–6 ▸. The values of *E*_1(⊥)_ shown in these figures are in agreement with those calculated using equation (7). In the crystal plane, Young’s modulus varies with direction, except in the Si(111) plane where it has a constant value of 169 GPa. The shear modulus and Poisson’s ratio involve two directions (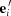, 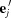). Results are shown in Figs. 3 ▸–6 ▸ for ‘in plane (∥)’ where 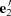 and 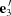 are in the (*h k l*) plane, and for ‘normal to plane (⊥)’ where 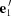 is fixed in the [*h k l*] direction and 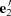 is in the (*h k l*) plane. Both shear modulus and Poisson’s ratio for Si(111) orientation are constant for the components in the plane or normal to the plane.

Attention should be paid to the order of the index in the Poisson’s ratio as, in general, ν_*ij*_ ≠ ν_*ji*_. This is the case when 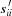 ≠ 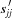, which can be easily checked by equations (8) or (10) and also numerical results. For instance, the Poisson’s ratio ν_12_ for Si(100) can be calculated by substituting the direction cosines of the vector 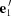 [100] and 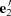 [0 cos(α) sin(α)] (see Fig. 2 ▸) in equation (10) as
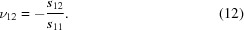
Poisson’s ratio ν_21_ is given by
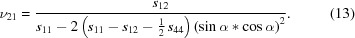
These two components of Poisson’s ratio are equal only at α = 0° or 90°. The Poisson’s ratio ν_12_ and ν_21_ for Si(100) are plotted *versus* angle α in Fig. 7 ▸. It is clear that for Si(100) Poisson’s ratio ν_12_ is, in general, not equal to ν_21_. For the shear modulus, the index *i*, *j* can be permuted as *G*_*ij*_ = *G*_*ji*_; this is clear from equation (11). Therefore, for the anisotropic elasticity of a silicon crystal, there are three independent components of Young’s modulus and the shear modulus, and six independent components of Poisson’s ratio.

**Figure 6:**
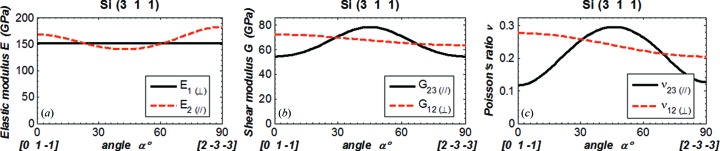
(*a*) Elastic modulus in the directions 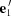 and 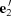. (*b*) Shear modulus and (*c*) Poisson’s ratio in the directions 12 and 23 for silicon (311). The vector 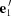 is fixed in the normal direction [311], and the vectors 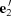 and 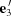 are in the crystal plane (311). The angle α is between the vectors 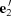 and [0 1 −1]/2^1/2^ in the crystal plane: 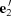
(α=0°) = [0 1 −1]/2^1/2^, 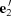
(α=90°) = [2 −3 −3]/(22)^1/2^.

As in the literature (Wortman & Evans, 1965 ▸; Kim *et al.*, 2001 ▸; Hopcroft *et al.*, 2010 ▸), we evaluate Young’s modulus, the shear modulus and Poisson’s ratio *versus* the angle α in the crystal plane at a reduced range of 0 to 90°. By symmetry, it is possible to deduce the results at any angle α larger than 90° from the results shown in Figs. 3 ▸–6 ▸. For instance, the results shown in Fig. 6 ▸ for Si(311) can be extended to the range 0–360° by using symmetry as depicted in Fig. 8 ▸, which was calculated for the angle α varying from 0 to 360°.

**Figure 7:**
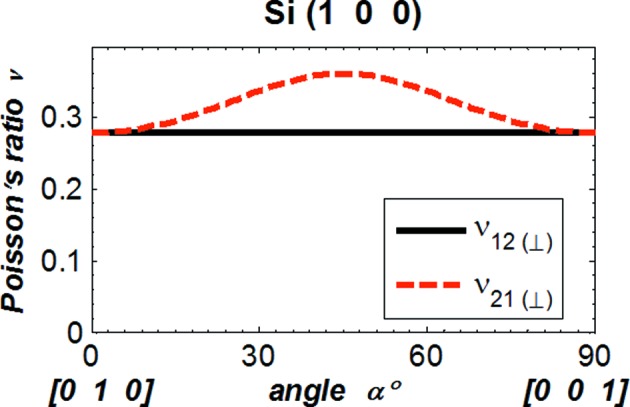
Poisson’s ratio ν_12_ and ν_21_ *versus* angle α between the vectors 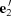 and [0 1 0]/2^1/2^ in the crystal plane for Si(100). See Fig. 2 ▸ for the definition of the coordinate system.

**Figure 8:**
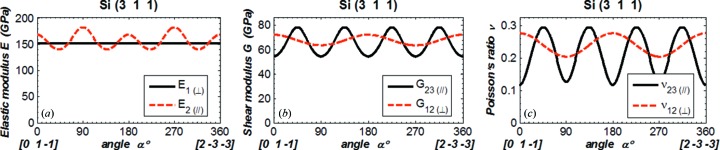
(*a*) Elastic modulus, (*b*) shear modulus, (*c*) Poisson’s ratio for silicon (311) *versus* the angle α varying from 0 to 360°.

Results shown in Figs. 3 ▸–5 ▸ and Fig. 7 ▸ for silicon crystal planes (100), (110), (111) have been compared with previously reported values (Wortman & Evans, 1965 ▸; Kim *et al.*, 2001 ▸; Hopcroft *et al.*, 2010 ▸) and summarized in Table 1 ▸. The present results are mostly in agreement with the literature; however, some discrepancies should be noted: for Si(100), the ‘normal to plane (⊥)’ component of Poisson’s ratio shown in Fig. 3(*c*) ▸ and by Wortman & Evans (1965 ▸) is ν_12_ where 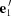 is fixed in the direction [100] and 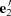 is varying in the plane (100) from direction [010] to [001]. But this ‘normal to plane (⊥)’ component of Poisson’s ratio ν_⊥_ for Si(100) shown by Kim *et al.* (2001 ▸) should be ν_21_ which is different from ν_12_ but in agreement with our results shown in Fig. 7 ▸. For Poisson’s ratio in the Si(111) plane (∥), the present work shows a value of ν_23(∥)_ = 0.262, in agreement with Kim *et al.* (2001 ▸) (ν**_∥_**), but Wortman & Evans (1965 ▸) presented a higher value of ν_∥_ = 0.358. For the shear modulus ‘normal to plane (⊥)’ for the Si(111) orientation, the present work gives a value of *G*_12(⊥)_ = 57.8 GPa, in agreement with Kim *et al.* (2001 ▸) (*G*_⊥_), but Wortman & Evans (1965 ▸) showed a smaller value of *G*_⊥_ = 47.0 GPa.

## 3. Mechanically bent X-ray optics   

Single-crystal silicon is the most commonly used material for the substrates of X-ray mirrors due to its interesting mechanical properties, and especially its excellent optical polishing quality. Dynamically bent mirrors in the Kirk­patrick–Baez (KB) configuration (Zhang *et al.*, 2010 ▸) offer a versatile approach for nano-focusing applications at the ESRF. Similarly, silicon Bragg polychromator crystals can be dynamically bent to elliptical shape to cover a wide photon energy range. In these two examples, the silicon crystal is bent to an ideal elliptical shape with an accuracy in the range of 10^−5^ for the ratio of slope error relative to the slope of the ideally bent shape. To achieve such a performance, the anisotropic elasticity must be taken into account in the simulation and shape optimization of the optics.

### 3.1. KB mirror profile optimization   

Nanofocusing of synchrotron X-ray beams using mirrors in the KB configuration can be achieved using reflective surfaces with an elliptical figure. For instance, the horizontal focusing mirror (HFM) of a multilayer-coated KB mirror device for the nano-imaging endstation ID22NI at the ESRF should have a radius of curvature in the range 11–30 m (*p* = 36 m, *q* = 83 mm, θ = 8 mrad). The use of dynamic bending technologies for this application allows the system to be optimized for operation over a large energy range (13–25 keV). One approach to achieve this highly aspheric shape is to use mechanical bender technology (Zhang *et al.*, 1998 ▸) based on elastic flexure hinges and variable-width mirrors (Zhang *et al.*, 2010 ▸).

**Figure 9:**
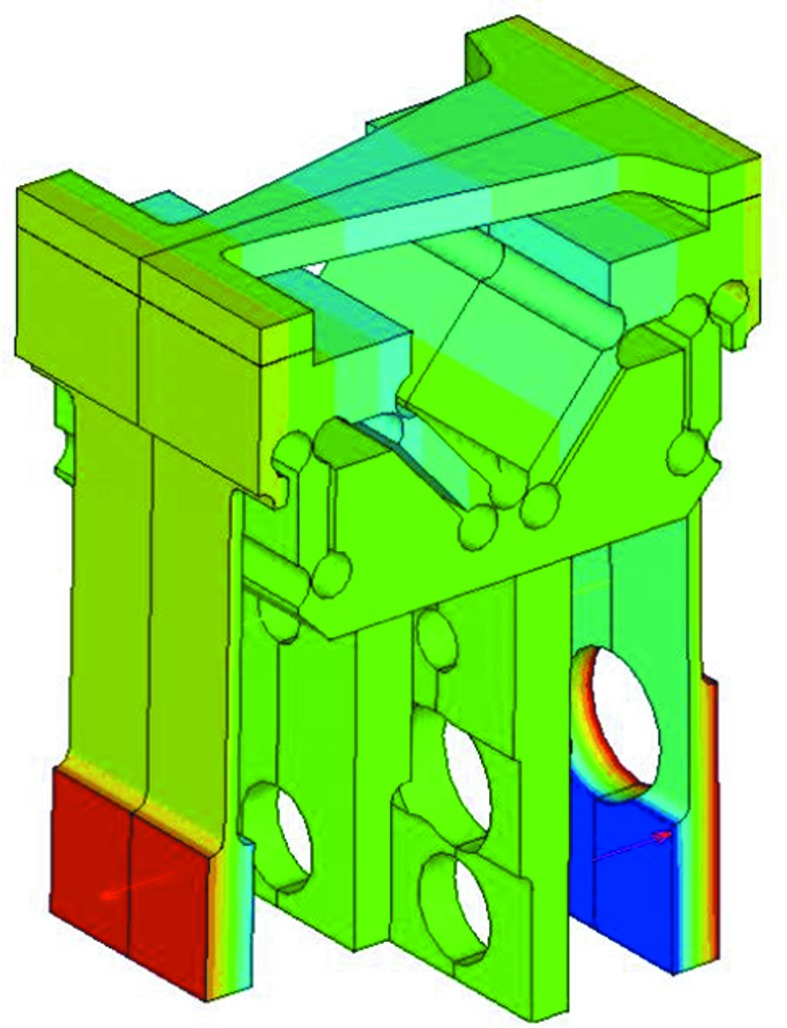
Finite-element model of the HFM mirror substrate and flexure bender assembly.

The application of two independent bending moments to the ends of the mirror substrate as in the ESRF bender design develops a linear variation of the moment along the mirror length. For the aspherical (elliptical) profile required, the radius of curvature *R*(*x*) of the bent substrate varies strongly with position *x* along the mirror length. The required variation over the useful mirror length for the ID22NI system is 58–32 m for the vertical focusing mirror (VFM) and 30–11 m for the HFM. The local slope of the bent substrate varies along the substrate length in the range of several mrad. Using the mechanical beam theory approximation, the local curvature, 1/*R*(*x*), can be calculated by
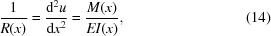
with
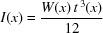
where *u* is the vertical displacement of the mirror, *x* is the mirror coordinate, *M*(*x*) is the local bending moments, and *E* and *I*(*x*) are, respectively, the elastic modulus and local moment of inertia of the mirror. *W*(*x*) and *t*(*x*) are the local width and thickness of the substrate, respectively. For a rectangular mirror, *I*(*x*) is constant and allows a third-order polynomial approximation to the ideal elliptical cylinder surface figure. For the mirror lengths and bending radius required for the ID22NI system, the figure/slope errors for this substrate geometry would be incompatible with the target performance. To overcome this limitation, a commonly applied approach at the ESRF is to use a trapezoidal profile for the mirrors, *i.e.* a linear variation in the substrate width, *W*(*x*), along the mirror. This allows correction of higher-order terms in the elliptical figure expansion. For improved correction of the figure errors it is necessary to use more complex width profiles (quadratic and beyond). For manufacturing simplicity the substrate thickness *t*(*x*) remains constant along the mirror length. By using equation (14), it is possible to define a variable profile as
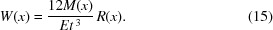
For the ID22NI HFM mirror it can be shown using finite-element modelling (FEM) that the slope error (differential slope between bent shape and ideal shape) with the profile defined analytically by equation (14) reaches 31 µrad RMS, which is much larger than the target requirement (<0.15 µrad). There are significant differences between FEM results on the mirror with the profile defined by equation (15) and the ideal ellipse. These are mainly due to the beam theory approximation in equation (14) which, unlike the FEM, does not take into account: (i) bender stiffness, (ii) anticlastic effects, and (iii) geometrical non-linear effects. The complete mirror and flexure bender assembly has been modelled in three-dimensions with FEM using *ANSYS* (Fig. 9 ▸). The silicon substrates were oriented such that the reflecting faces were parallel to crystal plane (110) with the [001] axis aligned along the mirror. This allows maximizing the ratio of the fracture toughness over the elastic modulus (Barrett *et al.*, 2011 ▸).

An iterative algorithm based on a fully parametrical finite-element model in *ANSYS* was used for the mirror width profile optimization (Zhang *et al.*, 2010 ▸), and reached the target requirement in performance for both horizontal and vertical focusing mirrors (HFM and VFM), as shown in Table 2 ▸. The mirror profiles were optimized for operation at 8 mrad of glancing angle (or photon energy at 17 keV). Using the optimized width profiles for 8 mrad as input to the FEM, it was also possible to calculate the expected slope errors over the full operating range of incidence angles (see Table 2 ▸). In the mirror width profile optimization by FEM, in addition to the above-mentioned three effects, we have also considered the influence of the adhesive bonding of the mirror to the flexure bender, the chamfer around the mirror, pre-loading springs and, of course, the anisotropic elasticity and crystal orientation.

From the optimized mirror width profiles, both HFM and VFM have been manufactured including the substrate machining and polishing, multilayer deposition, then assembled, and tested at the ESRF optical metrology laboratory. Measured results obtained are presented in Table 2 ▸. The measured slope error values are very close to the optimal theoretical values (Barrett *et al.*, 2011 ▸).

### 3.2. Crystal orientation and mirror axis   

The mirror width profile optimization was performed taking into account the anisotropic mechanical properties of the silicon crystal in the (110) crystallographic orientation for the mirror surface and axis [001] for the mirror meridional axis. This crystal orientation was chosen taking into account the anisotropy of the fracture behaviour of Si (Ebrahimi & Kalwani, 1999 ▸). By maximizing the fracture toughness along the planes perpendicular to the meridional direction and minimizing Young’s modulus along this same direction, the risk of brittle fracture during bending of the substrate can be reduced. For the convenience of FEM with *ANSYS*, the corresponding Cartesian coordinate system is oriented as: *x*-axis for the mirror meridional direction 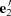 = [0 0 1], *y*-axis for the mirror sagittal direction 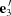 = [1 −1 0]/2^1/2^, and *z*-axis for the mirror normal direction 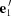 = [1 1 0]/2^1/2^. The stiffness matrix is given in the supporting information: 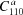, which is directly usable in *ANSYS*.

To show the importance of the correct consideration of the anisotropy of the silicon crystal, we consider two cases: (i) misaligned crystal orientation during mirror manufacturing, and (ii) mirror width profile optimization with constant isotropic mechanical properties.

#### 3.2.1. Misaligned crystal orientation during mirror manufacturing   

For the optical configuration of the HFM at photon energy 17 keV, the mirror width profile was optimized with the silicon crystal (110) aligned as described above. The calculated slope error (bent slope – ideal elliptical slope) is 0.09 µrad RMS. With this mirror width profile, we have simulated the cases where the silicon crystal is oriented in the following way:

(1) Crystal plane (110) and mirror axis in the direction [001]: as optimized.

(2) Crystal plane (110) and mirror axis in the crystal plane but α = 55° from the direction [001].

(3) Crystal plane (110) and mirror axis in the crystal plane but α = 90° from the direction [001].

(4) Crystal plane (100) and mirror axis in the direction [001].

(5) Crystal plane (111) and mirror axis in the direction [1 −1 0].

Results in RMS slope error, maximum bending stress and bending forces are given in Table 3 ▸. If two bending forces are fixed to 16 N as for the optimized case, the misaligned crystal orientation would lead to very significant performance degradation from 0.09 µrad to 162 µrad for case (2), *i.e.* crystal plane (110) and mirror axis in the crystal plane but 55° from direction [001]. By optimizing the bending forces for the misaligned cases, the slope error can be reduced but is still significantly higher than in the case of the correctly aligned crystal orientation. For example, in case (2), the slope error is 0.5 µrad instead of 0.09 µrad for the correctly aligned crystal. The bending forces are 21.4 N, 34% higher than the initially estimated 16 N for the correctly aligned crystal. To reach the same optical configuration, the bending forces and bending stress for a specified KB mirror are nearly proportional to Young’s modulus in the mirror axis (*E*_*x*_). To minimize the bending stress, the Si crystal orientation should be aligned in such a way that Young’s modulus is minimum (130 GPa) in the direction of the mirror meridional axis. Dynamical bending clearly offers the possibility to optimize the bending forces and greatly correct slope errors induced through any mis­alignment in the KB mirror.

#### 3.2.2. Mirror width profile optimization with constant isotropic mechanical properties   

For the same optical configuration of the HFM at photon energy 17 keV, if we use isotropic mechanical properties (for instance, *E* = 112.4 GPa, ν = 0.28, from Matweb) but the same bending forces (16 N), the optimized mirror width profile differs from the profile determined using anisotropic mechanical properties as defined in §3.2.1. Similarly, with this new mirror width profile, we have investigated the five cases listed in §3.2.1 and the results are summarized in Table 4 ▸. If the bending forces are fixed at 16 N, the slope error is very much higher than 0.09 µrad. By optimizing the bending forces for each case, the slope error can be reduced in the range 0.12–0.54 µrad, but is still significantly higher than in the case when the mirror width profile was optimized.

## 4. Thermal deformation of X-ray optics   

The thermal deformation modelling of silicon-based optics with anisotropic elasticity that we have initially performed concerns the liquid-nitrogen (LN_2_) cooled monochromator of the ESRF beamline ID06 (Zhang *et al.*, 2013 ▸). In this monochromator the silicon crystal (111) reflecting plane is used with the meridional axis aligned along the direction [1 −1 0]. For the convenience of FEM with *ANSYS*, the corresponding Cartesian coordinate system is oriented as: *x*-axis for the monochromator–crystal meridional direction 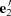 = [1 −1 0]/2^1/2^, *y*-axis for the mirror sagittal direction 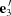 = [1 1 −2] /6^1/2^, and *z*-axis for the mirror normal direction 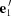 = [1 1 1] /3^1/2^. The stiffness matrix is given in the supporting information: 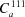, which is directly usable in *ANSYS*.

**Figure 10:**
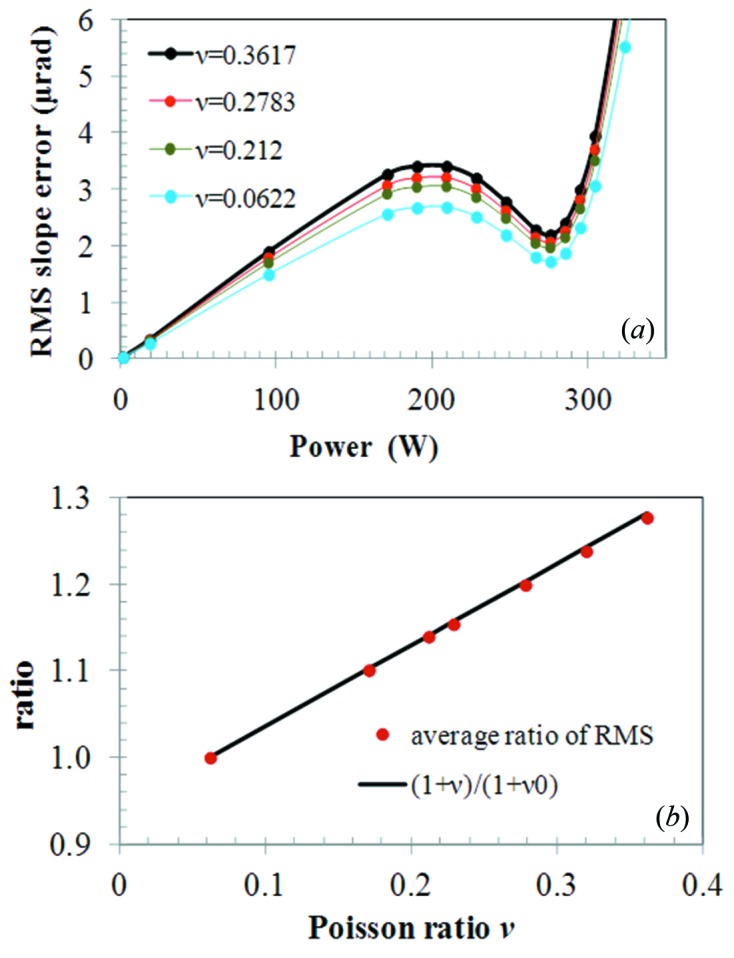
(*a*) RMS slope error over the whole footprint along the central axis on the crystal surface *versus* absorbed power. FEM was performed with isotropic mechanical properties and different Poisson’s ratio. (*b*) The average ratio of RMS slope (red points) and the ratio of (1 + ν)/(1 + ν_0_) (black line) *versus* Poisson’s ratio.

For a given absorbed power, the most influential material properties in the thermal deformation of X-ray optics are the thermal expansion coefficient α and the thermal conductivity *k*. For constant material properties, the thermal deformation is proportional to the ratio of these two parameters, α/*k*, and should be independent of the isotropic Young’s modulus. It is appropriate to note that both thermal expansion coefficient and thermal conductivity are second rank tensor properties which demonstrate isotropic behaviour in cubic crystals such as silicon. The influence of Poisson’s ratio on the thermal deformation was investigated. The value of Poisson’s ratio of silicon shown in Figs. 3 ▸–6 ▸ varies with crystal orientation in the range 0.0622–0.3617. We have performed a finite-element analysis of the LN_2_-cooled silicon crystal (Zhang *et al.*, 2013 ▸) with isotropic elastic properties and different Poisson’s ratio at first, and then with anisotropic elastic properties. The thermal deformation in terms of RMS slope error over the whole footprint along the central axis on the crystal surface is plotted *versus* absorbed power for Poisson’s ratio ν = 0.0622, 0.2120, 0.2783, 0.3617 in Fig. 10(*a*) ▸. These results show that the thermal deformation increases with Poisson’s ratio.

Taking the thermal deformation results at Poisson’s ratio ν_0_ = 0.0622 as reference, we have calculated the ratio of the RMS thermal slope at any Poisson’s ratio ν over that at Poisson’s ratio ν_0_ = 0.0622. This ratio of the RMS slope is almost constant for different absorbed power. The average of these ratios for different powers is plotted *versus* Poisson’s ratio (Fig. 10*b* ▸). The ratio (1 + ν)/(1 + ν_0_) is also plotted in Fig. 10(*b*) ▸. Results show that the thermal deformation of the monochromator crystal is a linear function of Poisson’s ratio, and the RMS slope error is proportional to the factor of 1 + ν. We can extend the relationship between the thermal deformation of X-ray optics and constant isotropic material properties as follows,
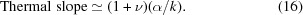
For a stable isotropic linear elastic material, Poisson’s ratio is in the range (−1, 0.5). Most materials have Poisson’s ratio values ranging between 0.0 and 0.5; ∼0.33 for many metals and nearly 0.5 for rubbers. Auxetic materials are those having a negative Poisson’s ratio, such as many polymer foams, cork, or magnetostrictive materials (such as Galfenol) in certain orientations. Some anisotropic materials have one or more Poisson’s ratios above 0.5 in some directions. The values for the materials used in X-ray optics (mirror substrates or monochromator crystals) are mostly in the range (0, 0.5). Therefore, the influence of Poisson’s ratio on the thermal deformation is less strong than the thermal conductivity and thermal expansion coefficient. This explains why the influences of Poisson’s ratio are commonly ignored in the evaluation of thermal deformation of X-ray optics.

**Figure 11:**
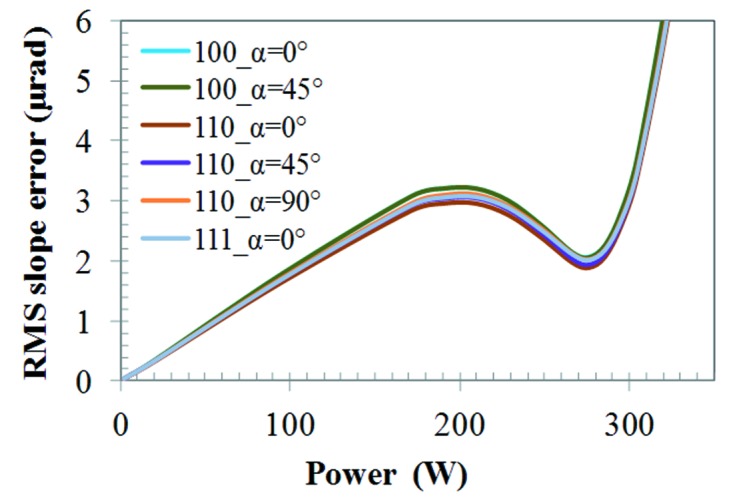
RMS slope error over the whole footprint along the central axis on the crystal surface *versus* absorbed power. FEM was performed with anisotropic mechanical properties and for different silicon crystal orientations.

We have made similar simulations to those shown in Fig. 10(*a*) ▸ but incorporating the anisotropic elastic properties of silicon for various crystal orientations. Results of thermal deformation in terms of RMS slope error *versus* absorbed power are depicted in Fig. 11 ▸. These results show that the thermal deformation depends slightly on the crystal orientation. As the meridional and sagittal directions are different crystal axes, the thermal deformation along these directions differs slightly, except in the case of Si(100) where the meridional and sagittal axes are equivalent. Among all these crystal orientations, the maximum thermal slope error *versus* absorbed power is for Si(100)_α=0° and Si(100)_α=45°, and the minimum is for Si(110)_α=0°. The difference between them is about 8.9%. Poisson’s ratio plotted in Figs. 3 ▸–6 ▸ varies from 0.0622 for Si(100) at the α=45° ‘in plane’ component ν_23(∥)_ and for Si(110) at the α=90° ‘normal to plane’ component ν_12(⊥)_ to 0.3617 for Si(110) at the α=0° ‘normal to plane’ component ν_12(⊥)_ and at the α = 90° ‘in plane’ component ν_23(∥)_. This leads to a ratio of (1 + 0.3617)/(1 + 0.0622) = 1.28, or possible difference in RMS slope of 28%.

**Figure 12:**
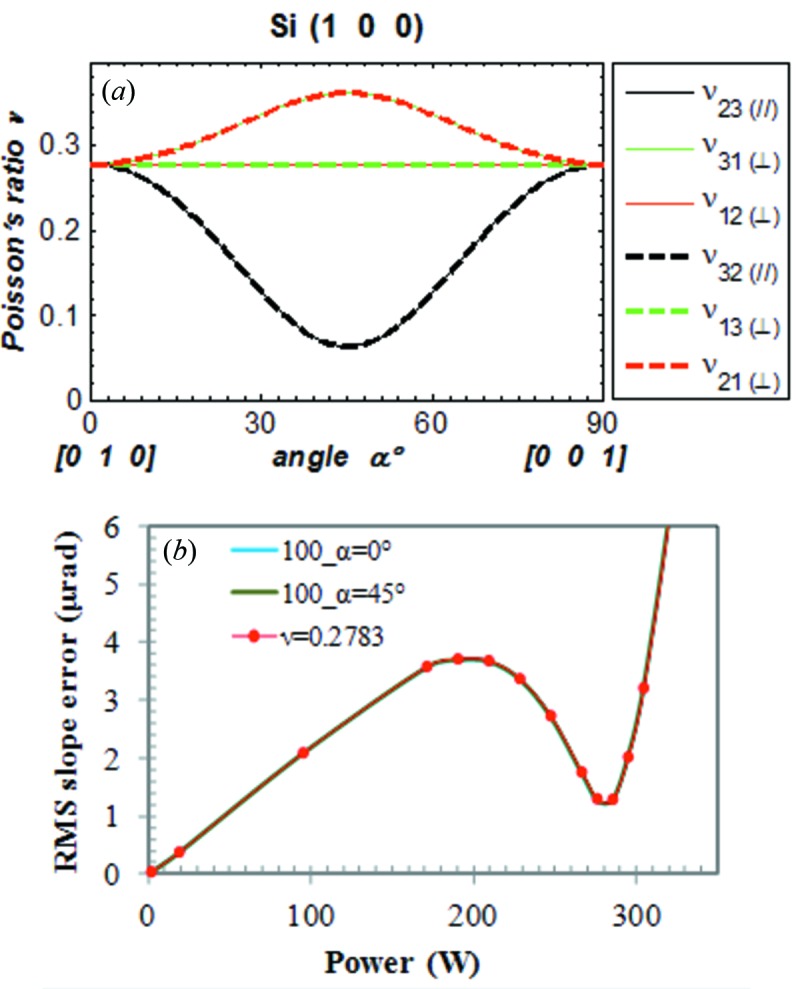
(*a*) The six components of Poisson’s ratio. (*b*) Thermal deformation in terms of RMS slope *versus* absorbed power for silicon (100)_α=0°, Si(100)_α=45° and isotropic and constant Poisson’s ratio ν = 0.2783.

**Figure 13:**
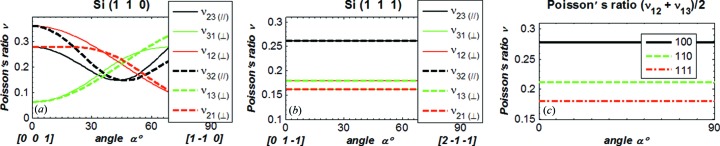
All six components of Poisson’s ratio for Si(110) (*a*) and Si(111) (*b*) *versus* the angle varying in the crystal plane. (*c*) The average ν_12_ and ν_13_ for Si(100), Si(110), Si(111).

**Figure 14:**
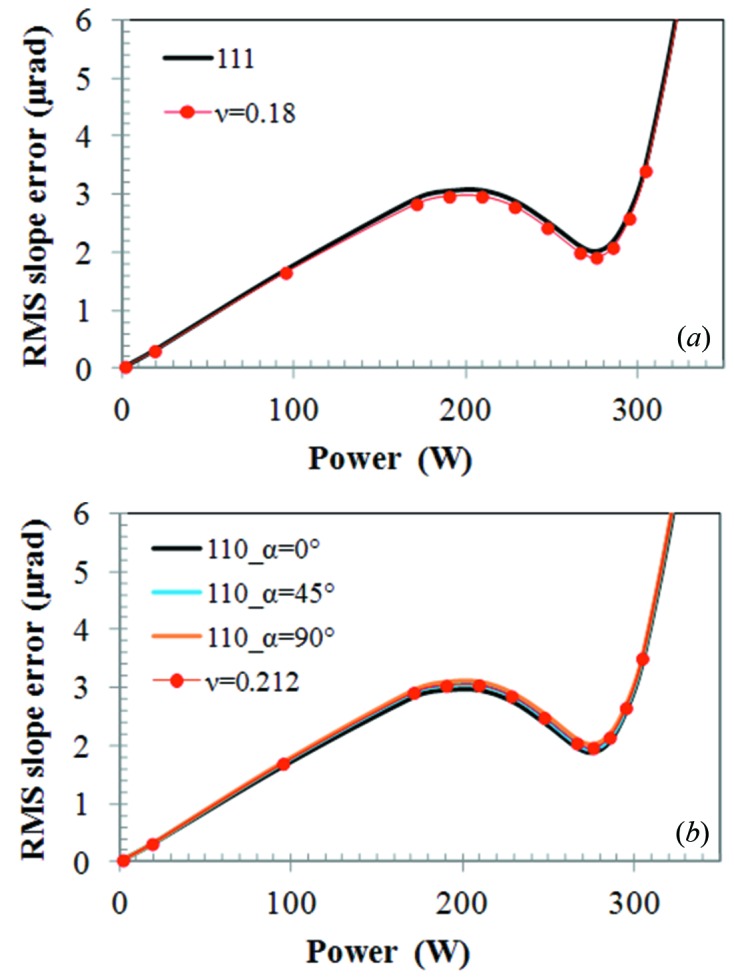
RMS slope error *versus* absorbed power (*a*) for anisotropic silicon Si(111) and for isotropic constant Poisson’s ratio 0.180 (ν_av_), and (*b*) for anisotropic silicon Si(110) at three angles in the crystal plane (α = 0°, 45°, 90°) and for isotropic constant Poisson’s ratio 0.212 (ν_av_).

For the anisotropic silicon crystal, there are six components of Poisson’s ratio (ν_*ij*_ with *i*, *j* = 1, 2, 3, *i* ≠ *j*). Fig. 12(*a*) ▸ shows the six components of Poisson’s ratio *versus* angle α as defined in Fig. 2 ▸ for Si(100). The ‘in plane’ components ν_23_ and ν_32_ are symmetrical and identical, but depend on the angle α. The ‘normal to plane’ components are not symmetrical, ν_13_ ≠ ν_31_ and ν_12_ ≠ ν_21_ as shown in §2. But we have ν_13_ = ν_12_ = 0.2783 independent of the angle α, and ν_31_ = ν_21_ varying with the angle α. The thermal deformation in terms of RMS slope error *versus* absorbed power for Si(100)_α=0°, Si(100)_α=45° and isotropic and constant Poisson’s ratio ν = 0.2783 is plotted in Fig. 12(*a*) ▸ and shows identical results. For Si(100)_α=0°, all six components of Poisson’s ratio are equal to 0.2783. However, for Si(100)_α=45°, ν_13_ = ν_12_ = 0.2783, ν_31_ = ν_21_ = 0.3617 and ν_23_ = ν_32_ = 0.0622. These suggest that thermal deformation depends mostly on the components of Poisson’s ratio ν_12_, ν_13_ or their average ν_av_ = (ν_13_ + ν_12_)/2, at least for Si(100). Note that the silicon crystal monochromator is oriented in such a way that the vector 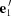 is normal to the crystal surface and 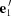 is along the meridional axis. The RMS thermal slope error is calculated from the derivative of the displacement normal to the crystal surface (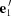) over the axis along the meridional direction (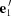). As an extension of the observations made for Si(100) described above, we have plotted the six components of Poisson’s ratio for Si(110) and Si(111) *versus* the angle α in Figs. 13(*a*) and 13(*b*) ▸. All six components of Poisson’s ratio for Si(110) vary strongly with α, including ν_12_ and ν_13_. However, the average ν_av_ = (ν_13_ + ν_12_)/2 is constant, 0.212. For all three crystal orientations Si(100), Si(110), Si(111), the average ν_av_ (Fig. 13 ▸ *c*) is independent of the angle α and equal to 0.212, 0.278 and 0.180, respectively. Then we plot the thermal deformation in terms of RMS slope error *versus* absorbed power for anisotropic silicon Si(111) and for isotropic constant Poisson’s ratio 0.180 (ν_av_) in Fig. 14(*a*) ▸, and for anisotropic silicon Si(110) at three angles in the crystal plane (α = 0°, 45°, 90°) and for isotropic constant Poisson’s ratio 0.212 (ν_av_) in Fig. 14(*b*) ▸. These results show that the thermal deformation of the LN_2_-cooled silicon crystal monochromator can be approximately simulated by using the isotropic constant Poisson’s ratio equal to the average of ν_12_ and ν_13_. The accuracy of this approximation is better than 1.2% for Si(100), 4.1% for Si(110) and 5.5% for Si(111). This approximation can be slightly improved by modifying the constant Poisson’s ratio according to the relation between the RMS slope and Poisson’s ratio in equation (15). For Si(111), for example, the average difference between the RMS slope calculated with anisotropic elasticity of Si(111) and an isotropic Poisson’s ratio ν_av_ = 0.18 is 3.08%. If we use a corrected Poisson’s ratio defined as follows, 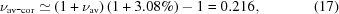
then this difference is reduced to 2.4%.

## 5. Summary   

The anisotropic elasticity of single-crystal silicon has been fully reviewed for arbitrary orientation of the crystal. A matrix-based computer algorithm is proposed for the calculation of the stiffness coefficient matrix, compliant coefficient matrix, Young’s modulus, shear modulus and Poisson’s ratio. It can be easily implemented in any numerical computing environment and programming language that include matrix analysis (Matlab and NumPy-Python examples are given in the supporting information). Analytical formulae to calculate Young’s modulus, the shear modulus and Poisson’s ratio are also summarized in this paper. Numerical values of Young’s modulus, the shear modulus and Poisson’s ratio have been compared with those in the literature, and have revealed discrepancies in some papers.

The anisotropic elasticity of single-crystal silicon has been used in the simulation of mechanical bent X-ray optics and thermal deformation of X-ray optics. For the mechanically bent X-ray optics, the silicon crystal orientation should be carefully taken into account both in optical design and manufacturing. Selection of the appropriate crystal orientation can lead to both an optimized performance and low mechanical bending stresses. A dynamic bending device allowing bending force optimization should be efficient in partially correcting the effects of crystal orientation alignment errors.

The thermal deformation of the crystal depends on Poisson’s ratio. For an isotropic constant Poisson’s ratio ν, the thermal deformation (RMS slope) is proportional to (1 + ν). For an anisotropic material with cubic crystal symmetry (such as silicon), the thermal deformation can be approximately simulated by using an isotropic constant Poisson’s ratio that is the average ν_av_ = (ν_13_ + ν_12_)/2, where direction 1 is normal to the crystal plane which is also the optic surface; the directions 2 and 3 are two normal orthogonal directions within the crystal plane. The average ν_av_ is independent of the direction in the crystal plane for Si(100), Si(110) and Si(111). Using this average Poisson’s ratio in the finite-element modelling of the thermal deformation of the X-ray optics leads to less than 5.5% of error in RMS slope in comparison with results from a full anisotropic analysis for Si(100), Si(110) and Si(111).

## Supplementary Material

Matrix-based computer algorithm and Matlab and Python codes for the calculation of anisotropic elasticity; and Stiffness matrix of silicon for crystal orientations (100), (110), (111), (311). DOI: 10.1107/S1600577514004962/ve5027sup1.pdf

## Figures and Tables

**Figure 4:**
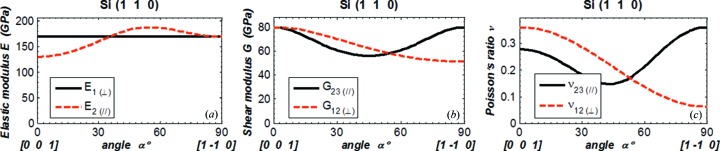
(*a*) Elastic modulus in the directions 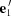 and 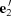. (*b*) Shear modulus and (*c*) Poisson’s ratio in the directions 12 and 23 for silicon (110). The vector 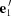 is fixed in the normal direction [110], and the vectors 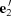 and 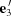 are in the crystal plane (110). The angle α is between the vectors 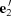 and [0 0 1] in the crystal plane: 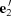
(*α* = 0°) = [0 0 1], 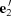
(*α* = 90°) = [1 −1 0]/2^1/2^.

**Figure 5:**
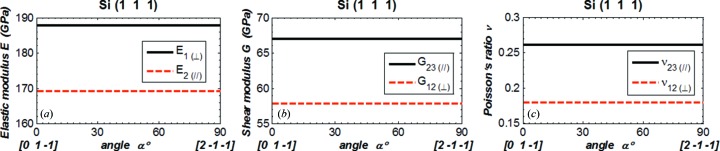
(*a*) Elastic modulus in the directions 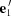 and 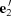. (*b*) Shear modulus and (*c*) Poisson’s ratio in the directions 12 and 23 for silicon (111). The vector 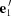 is fixed in the normal direction [111], and the vectors 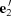 and 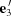 are in the crystal plane (111). The angle α is between the vectors 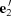 and [0 1 −1]/2^1/2^ in the crystal plane: 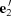
(α = 0°) = [0 1 −1]/2^1/2^, 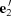
(α = 90°) = [2 −1 −1]/6^1/2^.

**Table 1:** Comparison between the present work and previously reported values (Wortman & Evans, 1965 ▸; Kim *et al.*, 2001 ▸; Hopcroft *et al.*, 2010 ▸) All results are mostly in agreement except three cases as indicated in footnotes †, ¶ and ††.

		Young’s modulus	Shear modulus	Poisson’s ratio
Si(100)	In-plane (∥)	Present, Wortman, Kim	Present, Wortman, Kim	Present, Wortman, Kim
	Normal to plane (⊥)	Present, Hopcroft, Equation (7*a*)	Present, Wortman, Kim	Present, Wortman, Kim†
Si(110)	In-plane (∥)	Present, Wortman, Kim‡	Present, Wortman, Kim‡	Present, Wortman, Kim‡
	Normal to plane (⊥)	Present, Hopcroft, Equation (7*b*)	Present, Wortman, Kim‡	Present, Wortman, Kim‡
Si(111)	In-plane (∥)	Present, Wortman, Kim§	Present, Wortman, Kim§	Present, Kim, Wortman¶
	Normal to plane (⊥)	Present, Hopcroft, Equation (7*c*)	Present, Kim, Wortman††	Present, Wortman, Kim§

† Poisson’s ratio ν_12_ for 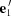 fixed in the direction [100] and 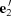 varying in the (100) plane, but ν_21_ is presented in Kim instead of ν_12_.

‡ Variation of direction (angle α) in plane: Kim from [0 0 1] to [−1 1 0], Present and Wortman from [0 0 1] to [1 −1 0].

§ Variation of direction (angle α) in plane: Kim from [1 −1 0] to [−1 −1 2], Present from [0 1 −1 ] to [2 −1 −1].

¶ Poisson’s ratio ν_∥_ in Si(111) plane: Present = Kim = 0.262, Wortman = 0.358.

†† Shear modulus *G*_⊥_ normal to plane for Si(111): Present = Kim = 57.8 GPa, Wortman = 47.0 GPa.

**Table 2:** Calculated by FEM and measured slope errors in RMS The mirror width profiles were optimized for operation at 8 mrad. The silicon substrates were oriented such that the reflecting faces were parallel to crystal plane (110) with the [001] axis aligned along the mirror.

Glancing angle			RMS slope error (µrad)	
θ (mrad)	*e*_ph_ (keV)	KB mirror	FEA	Measured
5.6	25	VFM	0.06	0.06
		HFM	0.11	0.11
8	17	VFM	0.08	0.09
		HFM	0.13	0.15
10.7	13	VFM	0.12	
		HFM	0.17	

**Table 3:** Calculated slope errors in RMS with different crystal plane and orientation using the mirror width profile optimized for the Si(110) plane and mirror axis [001]

	Si_*hkl*	Angle (°)	*F*_1_ (N)	*F*_2_ (N)	*E*_*x*_ (GPa)	RMS (µrad)	*S*_max_ (MPa)
Profile optimized for Si(110) plane, axis [001], fixed bending forces *F*_1_ and *F*_2_	110	0	16.00	16.00	130	0.09	42.5
	110	55	16.00	16.00	188	162	43.0
	110	90	16.00	16.00	169	123	45.1
	100	0	16.00	16.00	130	0.37	41.6
	111	0	16.00	16.00	169	121	41.8

Profile optimized for Si (110) plane, axis [001], optimized forces *F*_1_ and *F*_2_	110	0	16.00	16.00	130	0.09	42.5
	110	55	21.39	21.40	188	0.50	59.2
	110	90	19.97	19.54	169	0.18	56.2
	100	0	16.00	16.02	130	0.22	41.6
	111	0	19.72	19.64	169	0.24	52.5

**Table 4:** Calculated slope errors in RMS with different crystal plane and orientation using the mirror width profile optimized with isotropic material properties

	Si_*hkl*	Angle (°)	*F*_1_ (N)	*F*_2_ (N)	*E*_*x*_ (GPa)	RMS (µrad)	*S*_max_ (MPa)
Profile optimized for *E* = 112.4, ν = 0.28, fixed bending forces *F*_1_ and *F*_2_	110	0	16.00	16.00	130	75	34.0
	110	55	16.00	16.00	188	217	40.5
	110	90	16.00	16.00	169	184	37.5
	100	0	16.00	16.00	130	75	33.8
	111	0	16.00	16.00	169	181	39.0

Profile optimized for *E* = 112.4, ν = 0.28, optimized forces *F*_1_ and *F*_2_	10	0	18.17	17.97	130	0.12	38.7
	110	55	24.37	24.21	188	0.54	63.7
	110	90	22.84	22.06	169	0.24	53.5
	100	0	18.15	18.01	130	0.26	38.6
	111	0	22.48	22.16	169	0.23	55.8

## Footnotes

1 Supporting information for this paper is available from the IUCr electronic archives (Reference: VE5027).
