# Supplementary material for: Anisotropic elasticity of silicon and its application to the modelling of X-ray optics
Source: J Synchrotron Radiat. 2014 Apr 4;21(Pt 3):507–17. doi: 10.1107/S1600577514004962 (PMC4861878; doi:10.1107/S1600577514004962)
Supplement: Supplementary file 1 [file s-21-00507-sup1.pdf]

Matrix based computer algorithm and Matlab and Python codes for the calculation of anisotropic elasticity

For an arbitrary orientation of the cubic crystal with optical surface parallel to the (h k l) plane, the coordinate system (e_1, e_2, e_3) is defined by the surface normal [h k l] and two other orthogonal vectors in the crystal surface. The three normalized vectors could be

$$e_1 = [h\ k\ l] / \sqrt{h^2+k^2+l^2}\ ,\ e_2 = [0\ l\ {-k}] / \sqrt{k^2+l^2}\ ,\ e_3 = [\ (k^2+l^2)\ \ -h*k\ \ -h*l] / \sqrt{(k^2+l^2)*(h^2+k^2+l^2)} \qquad \text{(a)}$$

The formalism in (Wortman *et al.* 1965) can be used to calculate the stiffness coefficient matrix C_{hkl} and compliance matrix S_{hkl} for any silicon crystal plane. We have re-written this formalism in matrix form and coded in MatLab as follows:

$\mu = c_{11} - c_{12} - 2*c_{44}$;

Ce *= zeros(6,6) ;* ***Ce****(1,1)=μ;* ***Ce****(2,2)=μ;* ***Ce****(3,3)=μ;*

$\boldsymbol{M_0} = [e_1.*e_1;\ e_2.*e_2;\ e_3.*e_3;\ e_2.*e_3;\ e_1.*e_3;\ e_1.*e_2];$ $\quad \$\boldsymbol{M} = \boldsymbol{M_0} * \boldsymbol{M_0}';$

$\boldsymbol{C} = \boldsymbol{C_{100}} + \mu*\boldsymbol{M} - \boldsymbol{Ce};$

The compliant coefficient matrix $\boldsymbol{S}=\boldsymbol{C}^{-1}$ can be used to calculate Young's modulus E, shear modulus G and Poisson's ratio $\boldsymbol{v}$ as:

S = inv*(C)*;

E = [*1/S(1,1), 1/S(2,2), 1/S(3,3)*];

G = [*1/S(4,4), 1/S(5,5), 1/S(6,6)*];

v = - *S(1:3,1:3)./([1/S(1,1); 1/S(2,2); 1/S(3,3)]**ones*(1,3))*;

Where E and G are vectors 1x3, represents E = [E_{11}, E_{22}, E_{33}], G = [G_{23}, G_{13}, G_{12}]. Poisson's ratio matrix $\boldsymbol{v}$ is of dimension 3x3, there are 9 elements but only the 6 elements v_{12}, v_{13}, v_{21}, v_{23}, v_{31}, v_{32} are used. In general, the Poisson's ratio matrix is not symmetric: $v_{ij} \neq v_{ji}$. Attention should be paid to the order of index in the Poisson's ratio.

When using finite element software ANSYS, the 6x6 stiffness coefficient matrix $\boldsymbol{C}$ relates the terms ordered as {1, 2, 3, 12, 23, 31}, whereas for most published materials (Mason 1958, Wortman *et al.* 1965 for Silicon) the order is given as {1, 2, 3, 23, 31, 12}. This difference requires the $\boldsymbol{C}$ matrix terms to be converted to the expected format. It is more convenient to generate directly the stiffness coefficient matrix $\boldsymbol{C}$ in ANSYS convention by using following matrix $\boldsymbol{M_0}$:

$\boldsymbol{M_0} = \boldsymbol{M_{0a}} = [\ e_1.*e_1;\ e_2.*e_2;\ e_3.*e_3;\ e_1.*e_2;\ e_2.*e_3;\ e_1.*e_3];$

The coordinate system in finite element modelling is often defined with z-axis normal to the crystal optical surface, and x-axis and y-axis parallel to the crystal optical surface. In that case, the vectors given in Eq.(a) need to be permutated as $e_1 \rightarrow e_{3a}$, $e_2 \rightarrow e_{1a}$, $e_3 \rightarrow e_{2a}$, and the matrix M_0 is then:

$\boldsymbol{M_0} = M_{0a} = [\ e_{1a}.*e_{1a};\ e_{2a}.*e_{2a};\ e_{3a}.*e_{3a};\ e_{1a}.*e_{2a};\ e_{2a}.*e_{3a};\ e_{1a}.*e_{3a}];$

Matlab code for the calculation of anisotropic elasticity of silicon:

```
% Vectors e1,e2, e3 to be defined by user,  for instance
h=3; k=1;  l=1;
e1 =[h k l];  e1=e1/norm(e1);
e2 =[0 l -k];  e2=e2/norm(e2);
e3 =[(k^2+l^2) -h*k  -h*l];  e3=e3/norm(e3);

% Definition of Stiffness Matrix for Si(100)
c11 = 165.7 ; c12 = 63.9 ; c44 = 79.6;                 % GPa
C100=[c11 c12 c12 0 0 0; c12 c11 c12 0 0 0; c12 c12 c11 0 0 0; 0 0 0 c44 0 0; 0 0 0 0 c44 0; 0 0 0 0 0 c44];

mu= c11 -c12 -2*c44 ;
Ce = zeros(6,6) ;        Ce(1,1)= mu;      Ce(2,2)= mu;      Ce(3,3)= mu;
M0 = [ e1.* e1; e2.* e2; e3.* e3; e2.* e3; e1.* e3; e1.* e2];          % Common definition
% M0 = [ e1.* e1; e2.* e2; e3.* e3; e1.* e2; e2.* e3; e1.* e3];        % ANSYS definition
M= M0* M0';
C = C100 + mu *M - Ce;                                        % Stiffness Matrix (GPa)
S = inv(C);                                                   % Compliance coefficient Matrix
E = [1/S(1,1), 1/S(2,2), 1/S(3,3)];                           % Young's modulus (GPa)
G = [1/S(4,4), 1/S(5,5), 1/S(6,6)];                           % shear modulus (GPa)
nu = - S(1:3,1:3)./( [S(1,1); S(2,2); S(3,3)]*ones(1,3));     % Poisson's ratio Matrix
```

Python code using Numpy for the calculation of anisotropic elasticity of silicon:

```
import numpy as np
# Vectors e1,e2, e3 to be defined by user, for instance
h=3; k=1;  l=1;
e1 = np.array([h, k, l]);  e1 = e1/np.sqrt(np.dot(e1,e1))
e2 = np.array([0, l, -k]); e2 = e2/np.sqrt(np.dot(e2,e2))
e3 = np.array([(k*k+l*l), -h*k, -h*l]);  e3 = e3/np.sqrt(np.dot(e3,e3))

# Definition of Stiffness Matrix for Si(100)
c11 = 165.7 ; c12 = 63.9 ; c44 = 79.6   # GPa
C100= np.array( [ [c11, c12, c12, 0, 0, 0],[c12, c11, c12, 0, 0, 0],[c12, c12, c11, 0, 0, 0],[0, 0, 0, c44, 0, 
0],[0, 0, 0, 0,c44, 0],[0, 0, 0, 0, 0, c44]])

mu= c11 -c12 -2*c44 ;
# note that python indices start by zero
Ce=np.zeros((6,6)) ;  Ce[0,0] = mu  ; Ce[1,1] = mu  ; Ce[2,2] = mu
M0 = np.array( [e1*e1, e2*e2, e3*e3, e2*e3, e1*e3, e1*e2] )      # Common definition
#M0 = np.array( [e1*e1, e2*e2, e3*e3, e1*e2, e2*e3, e3*e1] )     # ANSYS definition
M = np.dot( M0,M0.transpose() )
C=C100+mu*M-Ce                                                  # Stiffness matrix (GPa)
S = np.linalg.inv(C)                                            # Compliance coefficient Matrix
E = [1/S[0,0], 1/S[1,1], 1/S[2,2]]                              # Young's modulus (GPa)
G = [1/S[3,3], 1/S[4,4], 1/S[5,5]]                              # shear modulus (GPa)
nu = -S[ 0:3,0:3 ] / np.array( [ [S[0,0],S[0,0],S[0,0]],[S[1,1],S[1,1],S[1,1]],[S[2,2],S[2,2],S[2,2]] ] )
                                                                # Poisson's ratio
```

Stiffness Matrix of silicon for crystal orientations (100), (110), (111), (311)

	Common	ANSYS convention
	$\{1\ \ 2\ \ 3\ \ 23\ \ 31\ \ 12\}$	$\{1\ \ 2\ \ 3\ \ 12\ \ 23\ \ 31\}$

Si (1 0 0)

$(e_1^{'}, e_2^{'}, e_3^{'})$ [1 0 0], [0 1 0], [0 0 1] [0 1 0], [0 0 1], [1 0 0]

$$C_{100} = \begin{bmatrix} 165.7 & 63.9 & 63.9 & & & \\ 63.9 & 165.7 & 63.9 & & & \\ 63.9 & 63.9 & 165.7 & & & \\ & & & 79.6 & & \\ & & & & 79.6 & \\ & & & & & 79.6 \end{bmatrix} \qquad C^a_{100} = \begin{bmatrix} 165.7 & 63.9 & 63.9 & & & \\ 63.9 & 165.7 & 63.9 & & & \\ 63.9 & 63.9 & 165.7 & & & \\ & & & 79.6 & & \\ & & & & 79.6 & \\ & & & & & 79.6 \end{bmatrix}$$

Si (1 1 0)

$(e_1^{'}, e_2^{'}, e_3^{'})$ [1 1 0], [0 0 1], [1 -1 0] [0 0 1], [1 -1 0], [1 1 0]

$$C_{110} = \begin{bmatrix} 194.4 & 63.9 & 35.2 & & & \\ 63.9 & 165.7 & 63.9 & & & \\ 35.2 & 63.9 & 194.4 & & & \\ & & & 79.6 & & \\ & & & & 50.9 & \\ & & & & & 79.6 \end{bmatrix} \qquad C^a_{110} = \begin{bmatrix} 165.7 & 63.9 & 63.9 & & & \\ 63.9 & 194.4 & 35.2 & & & \\ 63.9 & 35.2 & 194.4 & & & \\ & & & 79.6 & & \\ & & & & 50.9 & \\ & & & & & 79.6 \end{bmatrix}$$

Si (1 1 1)

$(e_1^{'}, e_2^{'}, e_3^{'})$ [1 1 1], [0 1 -1], [2 -1 -1] [0 1 -1], [2 -1 -1], [1 1 1]

$$C_{111} = \begin{bmatrix} 204 & 44.77 & 44.77 & 0 & 0 & 0 \\ 44.77 & 194.4 & 54.33 & 0 & 13.53 & 0 \\ 44.77 & 54.33 & 194.4 & 0 & -13.53 & 0 \\ 0 & 0 & 0 & 70.03 & 0 & 13.53 \\ 0 & 13.53 & -13.53 & 0 & 60.47 & 0 \\ 0 & 0 & 0 & 13.53 & 0 & 60.47 \end{bmatrix} \qquad C^a_{111} = \begin{bmatrix} 194.4 & 54.33 & 44.77 & 0 & -13.53 & 0 \\ 54.33 & 194.4 & 44.77 & 0 & 13.53 & 0 \\ 44.77 & 44.77 & 204 & 0 & 0 & 0 \\ 0 & 0 & 0 & 70.03 & 0 & 13.53 \\ 13.53 & -13.53 & 0 & 0 & 60.47 & 0 \\ 0 & 0 & 0 & 13.53 & 0 & 60.47 \end{bmatrix}$$

Si (3 1 1)

$(e_1^{'}, e_2^{'}, e_3^{'})$ [3 1 1], [0 1 -1], [2 -3 -3] [0 1 -1], [2 -3 -3], [3 1 1]

$$C_{311} = \begin{bmatrix} 183.7 & 58.68 & 51.09 & & -16.1 & \\ 58.68 & 194.4 & 40.42 & & 11.07 & \\ 51.09 & 40.42 & 202 & & 5.032 & \\ & & & 56.12 & & 11.07 \\ -16.1 & 11.07 & 5.032 & & 66.79 & \\ & & & 11.07 & & 74.38 \end{bmatrix} \qquad C^a_{311} = \begin{bmatrix} 194.4 & 40.42 & 58.68 & & 11.07 & \\ 40.42 & 202 & 51.09 & & 5.032 & \\ 58.68 & 51.09 & 183.7 & & -16.1 & \\ & & & 56.12 & & 11.07 \\ 11.07 & 5.032 & -16.1 & & 66.79 & \\ & & & 11.07 & & 74.38 \end{bmatrix}$$
